# Athletic Exercises

**Published:** 1881-02

**Authors:** 


### ATHLETIC EXERCISE.

YOUNG men are frequently to be met with who seem to have an excessive *pride* of muscular development; the great development is confined however to an undue enlargement of the muscles of the upper portions of the arms, at the expense of the muscles of the chest and of the general health.

The prominent physicians of cities have such patients frequently. They usually come to ask advice in regard to some unaccountable lung trouble. They point to their arms and ask how it is possible that a man who can swing a dumb bell weighing fifty pounds can have weak lungs, with spitting of blood and other unfavorable symptoms ? It is the easiest thing in the world, and the most natural result of such violent exercise. This "athletic exercise" craze has come to be a national calamity. It is injuring some of the best young men in the country. Every gymnasium in the country should be abolished, not that there are not some good features about them, but that the evil resulting from gymnastic exercises, as ordinarily practiced, does much more harm than good. The professional gymnast is always short lived ; the average term of such a life being less than six years. Were the amateur gymnast to exercise due caution at all times, and not strive to excel, such exercise might be almost as valuable as horse-back riding, which is best of all.

But this he seldom does, and the consequence usually is that in his efforts to perform some wonderful feat he does irreparable injury to some portion of his system.

A few years since I one day met a college friend who was on his way to a "health lifting" establishment, and he invited me to go with him. He harnessed himself into a lifting machine, and, raised nearly one thousand pounds amid the applause of those present. Thus encouraged, he went to another part of the room, and, having adjusted straps over his shoulders, lifted over a thousand pounds. I was invited to a little of this sort of amusement, but my boyhood's experience with the shovel and the hoe had given me a distaste for voluntary efforts of that sort.

After the lifting was over, my friend showed me the muscles of his arms, and spoke enthusiastically of the plan he had adopted as a promoter of health and strength. When my turn came to speak, I condemned the whole thing in unmeasured terms. I said that to continue that sort of straining for any considerable time would result in ruining his health. Of course I had just about as much influence over him in this matter as persons usually do who attempt to advise men against their fixed views.

It might have been two years later before I met my friend again; but how changed. He was "hobbling" down Broadway from Dey to Cortlandt street. It took him longer to go that distance than it would have required, two years before, to walk four blocks.

He said that he was completely broken down, and had little hope of ever being better. He said that his ambition to excel as a gymnast had ruined his health. This is a single case, but it is the representative of a thousand similar cases all over the country.

There is a certain point to which muscular exercise can be carried with safety. Just beyond that there is danger. A little applause, an ambition to do just a little more than some one else has done, is too frequently incentive enough to an effort that is disastrous.

It is said that a horse should live and be serviceable five times as long as the time necessary to his maturity.

This is six years; therefore a horse should be good and serviceable at thirty years of age. Owing to the extra strain that is daily being put on him by being overloaded a New York dray horse is worn out at twelve years. Men enjoy no immunity over these unfortunate dumb creatures when they subject themselves, though voluntarily, to a similar treatment.

---

Remedy for Poison Ivy.—Dr. S. A. Brown, U. S. N., states, in the *Medical Record*, that he has found a specific to the troublesome eruption produced by the poison oak or poison ivy so common in our woods and along old fences. This specific he finds in bromine, which he has used with unvarying success in at least forty cases. He uses the drug dissolved in olive oil, cosmoline, or glycerine, in the strength of from 10 to 20 drops of bromine to the ounce of oil, and rubs the mixture gently on the affected parts three or four times a day. The bromine is so volatile that the solution should be renewed every twenty-four hours. The eruption never extends after the first thorough application, and it promptly disappears within twenty-fours if the application is persisted in.

